# The MoxFo initiative—Mechanisms of action: Biomarkers in multiple sclerosis exercise studies

**DOI:** 10.1177/13524585231204453

**Published:** 2023-10-26

**Authors:** Sina C Rosenkranz, Michelle Ploughman, Lars G Hvid, P. Zimmer, K. Erickson, Jan-Patrick Stellmann, Diego Centonze, Manuel A Friese

**Affiliations:** Institute of Neuroimmunology and Multiple Sclerosis, Center for Molecular Neurobiology Hamburg, University Medical Center Hamburg-Eppendorf, Hamburg, Germany; Recovery & Performance Laboratory, Faculty of Medicine, Memorial University, St. John’s, NL, Canada; Exercise Biology, Department of Public Health, Aarhus University, Aarhus, Denmark; The Danish MS Hospitals in Ry and Haslev, Haslev, Denmark; Division of Performance and Health (Sports Medicine) Institute for Sport and Sport Science TU Dortmund University, Germany; Department of Psychology, University of Pittsburgh, Pittsburgh, PA, USA; AdventHealth Research Institute, Neuroscience, Orlando, FL, USA; PROFITH “PROmoting FITness and Health Through Physical Activity” Research Group, Sport and Health University Research Institute (iMUDS), Department of Physical and Sports Education, Faculty of Sport Sciences, University of Granada, Granada, Spain; Institute of Neuroimmunology and Multiple Sclerosis, Center for Molecular Neurobiology Hamburg, University Medical Center Hamburg-Eppendorf, Hamburg, Germany; APHM, Hopital de la Timone, CEMEREM, Marseille, France; Aix Marseille University, CNRS, CRMBM, UMR, Marseille, France; Department of Systems Medicine, Tor Vergata University, Rome, Italy; Unit of Neurology and Neurorehabilitation, IRCCS Neuromed, Pozzilli, Italy; Institute of Neuroimmunology and Multiple Sclerosis, Center for Molecular Neurobiology Hamburg, University Medical Center Hamburg-Eppendorf, Hamburg, Germany

**Keywords:** Multiple sclerosis, biomarkers, exercise, mechanism

## Abstract

**Background:**

As exercise exerts neurobiological and immunomodulatory effects, it might also act as a disease-modifying intervention in MS. However, a clear mechanistic link between exercise and disease-modifying effects in MS has yet to be established.

**Objective:**

Establish recommendations for future mechanistic exercise studies in MS.

**Methods:**

In regular meetings, members of the mechanisms of action group within the MoXFo (Moving eXercise research Forward in MS) initiative evaluated gaps of knowledge and discussed unmet needs in mechanistic MS research.

**Results:**

We concluded that biomarkers assessed in translational studies in humans and animals are essential to decipher the underlying mechanisms of exercise in MS. Consequently, we defined clear definitions of different types of biomarkers examined in MS exercise studies and operationalized their use to align with the research question and optimal testing time points. Furthermore, we provide key considerations to improve the rigor of translational studies and defined minimal reporting criteria for animal studies.

**Conclusion:**

The resulting recommendations are intended to improve the quality of future mechanistic exercise studies in MS and consequently lead to a better understanding of therapeutic approaches.

## Introduction

Regular exercise has beneficial effects on multiple organs and systems and has been proposed as a non-pharmaceutical disease-modifying intervention for several non-neurological and neurological disorders.^1–3^ Exercise has also been postulated as a disease-modifying intervention in MS^4–9^ as it not only confers clear benefits to physical capacity in MS patients, such as strength, endurance and balance, but also improves MS symptoms. However, there is debate regarding whether regular exercise dampens inflammation and protects from ongoing neuronal injury in MS. Results from several clinical trials, albeit of short duration, do not unequivocally support a positive effect of exercise on neurodegenerative processes in MS and other neurological diseases.^10–12^

In addition, a direct link between an exercise-induced mechanism and a disease-modifying effect in MS is currently lacking, although potential underlying mechanisms are plausible. Animal experiments have demonstrated multiple neuroprotective and anti-inflammatory effects of exercise^13–16^ and studies in healthy people as well as in MS patients suggest that exercise can exert structural and functional benefits to the central nervous system (CNS).^9,17–19^ However, large-numbered longitudinal studies in humans which clearly elucidate the mechanistic reprogramming of pathways and thereby clarifying how exercise may act as a disease-modifying intervention in MS are sparse. This is mainly due to inadequate methodology and funding of these often very cost- and time-intensive assessments. One of the obstacles of deciphering these pathways in humans is the inability to directly access neuropathological biomarkers. Studies examining exercise in animal models of MS in which tissue analysis, especially of the CNS, is accessible, may provide better insights about exercise research in MS patients.^20^ Furthermore, animal models are more uniform and allow for a higher control of variables which results in better consistency and potentially more rigorous results. However, similar to other research areas, exercise research in MS is hindered by the fact that there is still a gap in the alignment and translatability of preclinical MS exercise research to human studies. Translational exercise studies that examine effects in both humans with MS and biologically aligned animal models may help to decipher how exactly exercise could influence regenerative and anti-inflammatory events.

Currently, there are no guidelines or recommendations for performing studies that examine the underlying mechanisms of action for the beneficial effects of exercise in MS (in the following referred to “mechanisms of exercise”), which would help to align these studies and thereby improve the strength of the research results. In order to improve the rigor, coordination, consensus, guidance, and reproducibility of mechanistic exercise studies in MS and its translation into clinical practice, we formed an international consortium of MS-related experts in exercise mechanisms of action, which is part of the MoXFo (Moving eXercise research Forward in MS) initiative.^21^ We reviewed and critically evaluated research that addressed mechanisms of exercise in MS, identified knowledge gaps that needed to be addressed, and concluded that biomarkers used in translational approaches are essential to analyze the underlying mechanisms. We further defined recommendations for the assessment of biomarkers in humans and animals in order to accelerate future mechanistic exercise studies in MS and provided a checklist for reporting criteria of exercise studies in animal models of MS.

## Methods

The “Mechanisms of Action” subgroup (Chair: MAF; co-chair: SCR) consisted of eight international researchers with different expertise in mechanistic research in MS (exercise science, basic and clinical neuroscience, physiology, psychology, rehabilitation, translational neuroimmunology). The group met regularly via video conferences between 2020 and 2021. In the first phase, based on internal group discussions depending on the expertise of every group member, the group identified gaps in mechanistic research of exercise in MS. We concluded that biomarkers best represent underlying mechanisms but that clear definitions of biomarkers and guidelines regarding when to assess them are lacking. Furthermore, we agreed that coordinated translational approaches in humans and animals would result in the most comprehensive identification of exercise effects. We therefore defined three key points that are essential to improve future mechanistic exercise studies in MS in humans and animals: (1) definition of the corresponding biomarkers for the questions being asked; biomarkers reflecting neuroprotective or anti-inflammatory MS disease-modifying effects, biomarkers measuring changes to the proposed underlying exercise-induced mechanisms and biomarkers showing that the exercise intervention induced physiological effects on fitness; (2) identifying the optimal time points to collect biomarkers in short-term and long-term interventions and (3) pursuing translational approaches to clarify the disease-modifying mechanisms of exercise in MS. Three teams, based on the members’ expertise, were appointed in which existing and emerging study results of exercise research in MS but also other diseases were collected. Challenges and gaps were defined and recommendations for future research were generated. Results were then discussed with the entirety of the “Mechanisms of Action” subgroup with the goal to reach a consensus among all group members.

## Results

### Definition of the corresponding biomarkers for the questions being asked

First, biomarker outcomes need to be distinguished from clinical outcomes. Based on the International Classification of Functioning, Disability and Health (ICF) by the World Health Organization’s, outcomes can be matched to different categories. Clinical outcomes can be used to meet standards for regulatory approval of therapeutics (e.g. relapse rate or walking tests; *for details, see MoXFo “Outcomes” Group 3*). In contrast, biomarkers are biological parameters that serve as surrogate markers of physiological or pathophysiological processes.^22^ They refer to “body functions and body structures” of the ICF. Here again, for MS exercise studies, it is necessary to divide the biomarkers into three categories: (1) biomarkers, which reflect disease-modifying anti-inflammatory or neuroprotective effects in MS (“disease-modifying biomarkers”), (2) biomarkers that reflect the respective underlying exercise-induced molecular mechanism (“mechanistic biomarkers”), and (3) biomarkers which reflect established general physiological effects of exercise and are indicators of the extent (intensity and frequency) of exercise to ensure that a proper exercise intervention was performed (“manipulation check biomarkers”). Pairing the three categories of biomarkers with a clinical outcome of interest is likely important.

### Potential biomarkers to map disease-modifying effects of exercise

Biomarkers assessing possible disease-modifying effects in human MS exercise studies could be obtained from biological fluids such as blood or cerebrospinal fluid (CSF) (e.g. serum neurofilament light chain (sNFL))^23^ extracted from high-resolution structural images of the CNS (e.g. magnetic resonance imaging (MRI))^24^ or determined using functional neurophysiological evaluation of the CNS (e.g. evoked potentials^25^ or transcranial magnetic stimulation).^26^ The committee noted that most studies assessing biomarkers of potential disease-modifying effects of exercise in MS patients were exploratory in nature, rarely adequately powered and short in duration.^9,25^

We recommend ideally combining the primary clinical outcome with at least one biomarker that relates to the proposed underlying modification of the CNS to identify whether the degree of improvement in the clinical outcome is related to changes in corresponding biological processes. Some disease-modifying biomarkers are well-established in pharmaceutical studies but lack proper evaluation in exercise interventions. As a first step, exercise trials in MS should consider the use of promising disease-modifying biomarkers used in pharmaceutical trials that reflect inflammatory disease activity (e.g. number or volume of lesions per T_2_-weighted MRI scan or gadolinium-enhancing lesions per T_1_-weighted MRI scan), neurodegeneration (e.g. brain atrophy, peripapillary retinal nerve fiber layer thickness measured by optical coherence tomography (OCT) and sNFL) or both.^27,28^ Alignment of biomarkers across exercise and pharmaceutical interventions would add further insight into the underlying disease-modifying mechanisms, dynamics and impact of exercise in MS.

### Biomarkers which reflect the underlying molecular mechanisms for the benefits of exercise

There is likely a vast number of biological pathways that are influenced by exercise, and currently, there is no clear mechanism that explains the potential exercise-induced immunomodulatory and neuroprotective effects in MS. However, several mechanistic biomarkers exist that have been examined in MS exercise studies, which indicate potential mechanisms such as neurotrophins,^17,29,30^ neurotoxic proteins,^23^ brain perfusion,^31^ myokines^30^ cytokines^29,30^ or immune cell subset population.^32^ We recommend including one or more mechanistic biomarkers to elucidate the mechanism for the proposed exercise-mediated disease-modifying effects. It is critical to discuss if biomarkers detected in the blood are also present in the CNS (e.g. by crossing the blood-brain barrier) or whether these molecules may at least partly mediate the disease-modifying effects. If this is not known, investigating the biomarker’s CSF levels or translational studies in animal models of MS are an attractive solution. Mechanistic exercise-related biomarkers examined in healthy participants^33,34^ and other neurological disorders could provide insights about potential biomarker candidates.

Furthermore, biomarkers of well-established exercise-induced pathways with effects on the CNS should be considered to include, for example, the exercise-induced myokine irisin, which can be detected in the blood. Irisin induces the expression of brain-derived neurotrophic factors and other potential neuroprotective genes in the hippocampus.^35^ In addition, a general understanding of exercise-induced dose–response effects are lacking, and thus also the potential associations between the exercise intensity or frequency and the biomarker levels. Such knowledge would be helpful in prescribing an accurate “personalized” exercise program. In order to include the most promising disease-modifying and mechanistic biomarkers in MS exercise studies, considerable planning, resources, and collaborations are required. Future research would be strengthened by harmonizing a series of plausible biomarkers across studies in order to consolidate data and draw definitive conclusions.

### Biomarkers to map the exercise intensity, frequency and general gain in physical fitness

In exercise research, it is important to determine the extent (intensity and frequency) and the adherence to the exercise intervention, which, similar to many pharmaceuticals, will vary from person to person. Exercise has clear physiological effects, for example, on heart and muscle, which can be easily gauged by physical examination (e.g. heart rate, maximal oxygen consumption (VO_2max_), maximum work load (P_max_), energy consumption, muscle strength), or extracted and assayed from body fluids (e.g. lactate, myokines) or tissue (e.g. muscle biopsy). Despite the availability of such measures, it is surprising to observe that some MS exercise studies did not include a biomarker that would indicate the extent of exercise or the gain in physical fitness.^36^ We concluded that exercise studies in MS should not proceed without at least one well-validated biomarker as a manipulation check measuring the extent or intensity of exercise or the gain in physical fitness, ideally both.

In preclinical research, it could be more challenging to assess biomarkers that map the extent of exercise and the gain of physical fitness. Such biomarkers may require invasive heart rate monitoring, tissue sampling, or frequent blood draws that could potentially compromise the animal’s well-being. This issue may explain why most of the MS exercise studies in animals lack such biomarkers. Despite such barriers, repeated measurement of body weight or endpoint analysis of body fat or muscle fiber types is feasible. We concluded that in animal studies employing an exercise intervention, at least one manipulation check biomarker should be included.

### Identifying the optimal time points to collect biomarkers in exercise studies in MS

After choosing the most appropriate biomarkers, the next most important decision relates to the optimal time point to collect the biomarker of interest. Studies typically examine biomarkers after an acute bout of exercise, while others examine biomarkers after long exercise durations in MS patients. There are only a few publications investigating the short- and long-term changes of a biomarker within one study.^30,37^

Here, we define short-term changes as acute effects that are directly detectable during or after one session of exercise and long-term adaptations that are induced by repeated sessions of exercise. It is important to know the biological dynamics and kinetics of the assessed biomarker to measure it at the appropriate time period, as some biomarkers change with the time interval that has elapsed in relation to the session or complete intervention. Changes in neurotrophins, cytokines, or immune cells are usually detectable during the intervention,^23,30,38^ however, long-term changes are important to understand their potential effects on the CNS and the neuroimmune axis. For instance, biomarkers depicting disease-modifying effects, such as structural MRI, may require several months or years of tracking to confirm exercise-induced changes. Recurring measurements are needed to identify the dynamics of biomarkers over time, as short-term effects might be misleading.^39^

The appropriate timepoint to measure a biomarker depends on the research questions and biological plausibility. Studies employing disease-modifying biomarkers and mechanistic biomarkers investigating the long-term effects of exercise should ideally include frequent measurements. Aligning with pharmaceutical trials, we recommend at least the following three timepoints for disease-modifying and mechanistic biomarkers: (1) baseline, (2) directly after termination of the intervention study period, and (3) follow-up timepoint after stopping the intervention. We further advise adding short-term measurements for the mechanistic biomarkers to compare the short- and long-term changes: (1) before the first intervention; these samples could also be used as baseline samples for long-term changes and (2) directly after the first session. Manipulation check biomarkers indicating the intensity of the intervention (e.g. heart rate, P_max_) should be monitored during the sessions. Manipulation check biomarkers indicating a gain of physical fitness (e.g. VO_2max_) should at least be determined at (1) baseline and (2) after the termination of the intervention study period. Although the collection of data at these timepoints might seem obvious, not all studies include these timepoints, especially the follow-up timepoint after stopping the intervention.

It is of utmost importance that studies report the time periods between the initiation and cessation of the intervention and the assessments of the biomarkers. Another important factor that needs to be taken into account when assessing biomarkers is whether the values might vary in relation to the time of the day. Some biomarkers (e.g. the numbers of immune cell subpopulations in the blood) fluctuate during the day, which needs to be considered in terms of collection protocols and interpretation of results. We recommend assessing biomarkers having diurnal fluctuations at the same time of day throughout the study. Relapses and exacerbations might also affect biomarker levels; hence, an additional time point after remission should be considered. The assessments during relapses or exacerbations should be reported and separately analyzed.

Timing of measurement of the biomarker is as important in animal models as in clinical studies. Exercise studies in MS animal models typically focus on long-term changes.^16^ Again, logistical barriers and concerns for animal welfare may preclude multiple invasive tests. We suggest that animal studies include two cohorts: one for acute effects and one for long-term effects. Because the lifespan of rodents and other animal models is much shorter than that of humans, it is important to design studies that replicate human exercise patterns. Based on animal exercise studies, which show that most of the long-term effects on the CNS occur after a period of 4 weeks,^14^ we recommend this as a minimum period for animals if addressing long-term effects. Interestingly, when adjusted to the human life span, this would be comparable to an intervention in humans of approximately 3 years. We advise assessing short-term and long-term changes of the respective mechanistic and disease-modifying biomarkers in animal exercise MS studies at the same timepoints as recommended above for humans.

### Pursuing translational approaches to foster the disease-modifying mechanisms of exercise in MS

As outlined, biomarker assessment in exercise MS studies would ideally be conducted in humans and animal models in parallel. Tissue biomarkers, which might explain the best mechanisms of action in the CNS, are more difficult to obtain in humans and more easily accessible in animals. Biomarkers such as blood, MRI, OCT, and electrophysiological assessments are possible in both humans and mice and could be used for parallel assessments.

However, there are a few points to consider when planning translational exercise studies. First, exercise behavior is quite different in rodents compared to humans. For instance, voluntary running in a running wheel reflects the natural behavior of the animals and is sporadically performed at night. Voluntary running is still used as the gold standard of exercise in animal models. Since it reflects the animal’s physiological behavior, it may be comparable to a human study investigating the effect of 7500 steps/day. On the other hand, swimming, treadmills, and some of the resistance interventions are provided for fixed durations but increase the stress levels of the animals.^40–42^

An additional problem is that aerobic exercise and high-intensity exercise are not well defined in animal models as they depend on the heart rate, which is technically challenging and rarely measured. Performing exercise is complicated by the fact that, in most MS animal models, the severity of disability impedes the animal’s ability to exercise. Although using models with less impairment may permit exercise interventions, changes in only mild deficits may be difficult to detect. Such challenges often lead researchers to implement exercise interventions prophylactically, either before symptoms occur or even before the CNS inflammation is induced, mimicking a prevention rather than a treatment strategy.^13,43^ Choosing the optimal time point to deliver the exercise should be clearly linked to the research questions (i.e. prevention versus treatment). Another challenge is that the animal models of MS may not ideally represent human pathophysiology. We therefore recommend that researchers provide a rationale for choosing their animal model. According to guidelines for animal exercise for cardiovascular studies,^44,45^ we defined criteria for improving the experimental design and reporting standards to improve the quality of exercise intervention studies in animal models of MS (Table 1). A future goal of the MoXFo group is to additionally define reporting criteria for exercise studies in humans.

**Table 1.** Recommended minimal reporting criteria and additional reporting criteria for MS exercise studies in animal models.

**Method*s*:** *Minimal reporting* - Species of animals - Number of females and males (balanced sex ratio recommended) - Age of animals - Calculation of number of animals (e.g. power analysis) - Randomization and blinding procedure - Inclusion and exclusion criteria for analysis, per protocol or intention to treat analysis - Number of animals for exercise and control groups (in total, but also experimental units (animals per cage)) - Detailed set-up of intervention (e.g. wheel size, treadmill company, water temperature, size of pool) - Procedure of intervention (one by one, all at the same time) - Total duration of intervention - Time of day at which intervention was performed - Criteria for stopping an intervention prematurely - Time between intervention and assessment of biomarkers - Ethical statement *Additional reporting* - Housing - Animal study registry prior to the start of the study
**Results:** *Minimal reporting* - Explanation of sort of intervention based on the research question, forced vs voluntary (e.g. swimming, voluntary running, treadmill) - Explanation of alignment to human study (e.g. prevention vs intervention) - Explanation of selected disease-modifying and mechanistic biomarkers and time points - Explanation of selected primary outcome measure - Frequency and duration of each bout of session - Drop outs - Weight of all animals at least 1×/week - Volume of intervention per animal (e.g. distance per day or session) - Manipulation check biomarker (e.g. heart rate, VO_2max_, Watt, muscle loading, body weight, muscle mass, contractile muscle properties, body fat determination, muscle staining for muscle fiber type switch (e.g. cytochrome c oxidase and succinate dehydrogenase), hippocampus staining for marker of neurogenesis, qPCR, e.g. *Ppargc1a* of muscle at the end of the study in exercise and control group) *Additional reporting* - Activity level outside the intervention - Food consumption during the study - Stress hormones (e.g. cortisol levels before and after the intervention)

**Figure 1.**
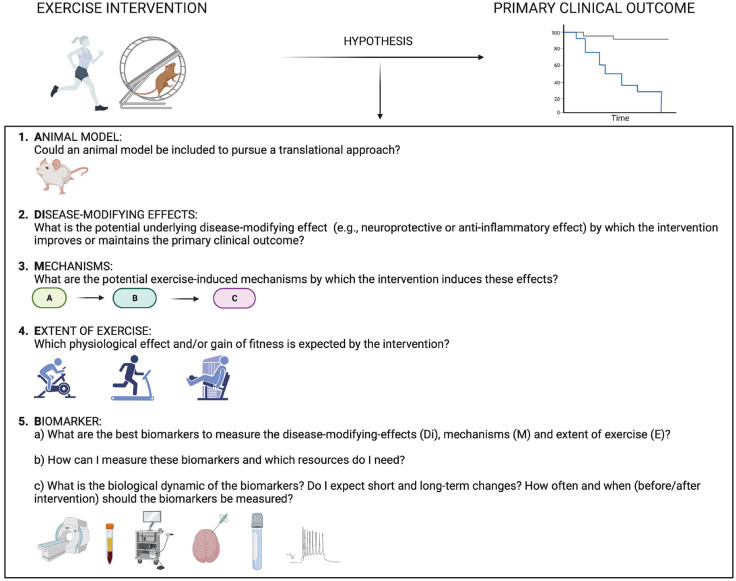
**Mechanistic key questions investigators should ask when designing their exercise studies in MS.** The flowchart shows a structured approach to uncover the underlying mechanisms of the expected primary clinical outcome of the exercise intervention following the ADiMEB checklist.

In summary, we here provide recommendations for performing mechanistic exercise studies in MS, which could help to align these studies and thereby improve the understanding of potential therapeutic approaches. Figure 1 depicts key questions that investigators should ask when designing a hypothesis-driven mechanistic exercise study in MS. These questions deal with the **a**nimal model, the **di**sease-modifying effect, the underlying exercise-induced **m**echanism, the **e**xtent of exercise, and the corresponding **b**iomarkers (**ADiMEB** checklist).

## Discussion

Here, we aimed to establish recommendations for future mechanistic MS exercise studies that help to implement standardized readouts and, consequently, a better understanding of the mechanisms of action for the beneficial effects of exercise in MS. We conclude that biomarkers assessed in human and animal studies in parallel represent the best way to decipher the underlying mechanisms. We defined definitions of different types of biomarkers: Biomarkers that reflect (1) the expected disease-modifying effect, (2) the proposed underlying mechanisms, and (3) a manipulation check for the intervention. These should always be combined with the primary clinical outcome, should align clearly with the research questions, and should ideally be predefined and validated. The time points of biomarker assessment should strategically map potential short- and long-term effects of exercise considering the biological dynamics of the biomarker. To facilitate these considerations, we provide key questions researchers should ask when designing mechanistic exercise studies in MS. Translational approaches that carefully align the exercise intervention and key biomarkers between human and animal studies will advance the understanding of the mechanisms of exercise in MS. To support this, we also defined minimal reporting criteria for animal studies.

Whereas implementing the proposed settings in upcoming clinical exercise studies in MS is associated with a minimal higher burden for the participating patients due to increased time expenditure and more frequent assessments, there are still some challenges. It requires a wide range of equipment (e.g. MRI, OCT, and laboratory) and personnel with different expertise, which is associated with higher expenses. However, collaborations between facilities as well as extended funding, would enable this comprehensive approach. While research results from different diseases have been considered in the evaluation, we acknowledge that the proposed recommendations are based on the research and appraisal of the committee members and not acquired by a structured approach (e.g. Delphi).
